# 334. The devil is in the details: Systematic evaluation of antimicrobial allergy at an academic medical center in Japan

**DOI:** 10.1093/ofid/ofad500.405

**Published:** 2023-11-27

**Authors:** Maho Adachi Katayama, Koh Okamoto, Koh Okamoto, Kyotaro Kawase, Ryo Yamaguchi, Satoshi Kitaura, Kohei Ukai, Toshiki Miwa, Yuji Wakimoto, Tatsunori Oyabu, Daisuke Jubishi, Hideki Hashimoto, Sohei Harada, Shu Okugawa, Takeya Tsutsumi

**Affiliations:** The University of Tokyo Hospital, Bunkyo-ku, Tokyo, Japan; The University of Tokyo Hospital, Bunkyo-ku, Tokyo, Japan; The University of Tokyo Hospital, Bunkyo-ku, Tokyo, Japan; The University of Tokyo Hospital, Bunkyo-ku, Tokyo, Japan; The University of Tokyo Hospital, Bunkyo-ku, Tokyo, Japan; National Institute of Infectious Diseases, Shinjuku-ku, Tokyo, Japan; The University of Tokyo Hospital, Bunkyo-ku, Tokyo, Japan; University of Tokyo Hospital, Tokyo, Tokyo, Japan; The University of Tokyo Hospital, Bunkyo-ku, Tokyo, Japan; The University of Tokyo Hospital, Bunkyo-ku, Tokyo, Japan; The University of Tokyo Hospital, Bunkyo-ku, Tokyo, Japan; The University of Tokyo Hospital, Bunkyo-ku, Tokyo, Japan; The University of Tokyo Hospital, Bunkyo-ku, Tokyo, Japan; The University of Tokyo Hospital, Bunkyo-ku, Tokyo, Japan; The University of Tokyo Hospital, Bunkyo-ku, Tokyo, Japan

## Abstract

**Background:**

Antimicrobial allergy is common and encountered by healthcare providers. Inaccurate or incomplete information on antimicrobial allergy leads to inappropriate antimicrobial selection. In the electronic health record (EHR), antimicrobial allergy is not only documented as free text but also listed in the EHR allergy section (EHR-AS). However, its quality has not been well studied.

**Methods:**

This was an observational study at an academic medical center in Tokyo, Japan. We included hospitalized patients who received infectious disease consultations from April 2022 to March 2023. Antimicrobial allergy was assessed through structured interview and review of allergies documented free-text in the provider’s note in the EHR (referred to systematic assessment hereafter). We evaluated the potential for de-labeling. We then compared it with the information in the EHR-AS.

**Results:**

A total of 1,179 patients were included. Systematic assessment revealed 148 (148/1179, 13%) patients had allergy histories related to 214 antimicrobials (Table). Of these, 38 patients (38/148, 26%) had a history related to more than one antimicrobial. Beta-lactam antibiotics accounted for 58% (125/214). Of 214 antimicrobials, allergy histories were documented in 70%, 53%, and 41% of notes written by primary team physicians, nurses, and pharmacists, respectively. In total, 71% (152/214) of antimicrobials in 107 patients found through systematic assessment could have been candidates for de-labeling; 21 for direct de-labeling, 19 for supervised oral rechallenge, and 112 for skin testing followed by oral rechallenge. The remaining 62 antimicrobials were deemed not candidates for de-labeling because of their high-risk features. Out of 214 antimicrobials, 126 (59%) were listed in the EHR-AS and the details were NOT described for 50 antimicrobials (50/126, 40%).

**Figure:**
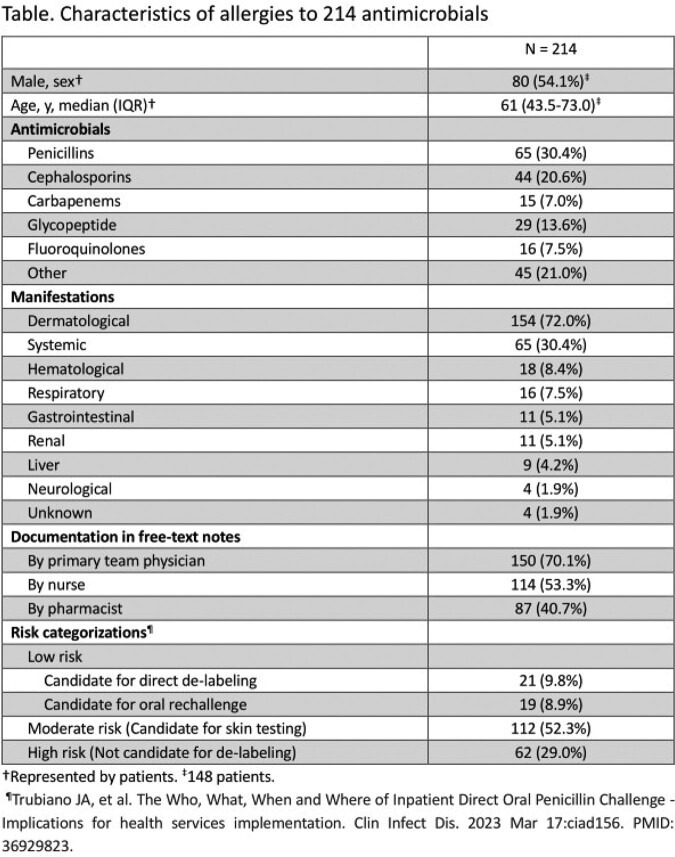

**Conclusion:**

While a majority of antimicrobial allergies could have been candidates for de-labeling when evaluated thoroughly, much fewer allergies were listed in the EHR-AS, often without details. Standardizing the evaluation of antimicrobial allergy and entry to the EHR-AS may facilitate selection of appropriate antimicrobials.

**Disclosures:**

**Satoshi Kitaura, MD, PhD**, FUJIFILM Toyama Chemical Co. Ltd.: S.K., during graduate school studies, conducted antiviral research involving favipiravir, which was provided by FUJIFILM Toyama Chemical Co. Ltd.

